# Image-based many-language programming language identification

**DOI:** 10.7717/peerj-cs.631

**Published:** 2021-07-23

**Authors:** Francesca Del Bonifro, Maurizio Gabbrielli, Antonio Lategano, Stefano Zacchiroli

**Affiliations:** 1 University of Bologna, Bologna, Italy; 2 Université de Paris, Paris, France; 3 Inria, Paris, France

**Keywords:** Programming language identification, Image recognition, Machine learning, Deep learning, Convolutional neural network, Code snippet, Source code

## Abstract

Programming language identification (PLI) is a common need in automatic program comprehension as well as a prerequisite for deeper forms of code understanding. Image-based approaches to PLI have recently emerged and are appealing due to their applicability to code screenshots and programming video tutorials. However, they remain limited to the recognition of a small amount of programming languages (up to 10 languages in the literature). We show that it is possible to perform image-based PLI on a large number of programming languages (up to 149 in our experiments) with high (92%) precision and recall, using convolutional neural networks (CNNs) and transfer learning, starting from readily-available pretrained CNNs. Results were obtained on a large real-world dataset of 300,000 code snippets extracted from popular GitHub repositories. By scrambling specific character classes and comparing identification performances we also show that the characters that contribute the most to the visual recognizability of programming languages are symbols (e.g., punctuation, mathematical operators and parentheses), followed by alphabetic characters, with digits and indentation having a negligible impact.

## Introduction

Programming Language Identification (PLI)—also referred to as Source Code Classification (SCC) in the literature—is the problem of identifying the programming language in which a given source code file, or just a short code snippet, is written in. PLI is a common preliminary need in automated program comprehension and also a relevant practical problem for both practitioners and researchers, with important applications in programming trend analysis (Chen et al., 2005), mining software repositories (Mockus, 2009; Caneill, Germn & Zacchiroli, 2017), source code indexing, and code search (Gallardo-Valencia & Sim, 2009; Kononenko et al., 2014).

Traditionally, PLI has been implemented in effective tools  (Wheeler, 2001; Danial, 2006; GitHub, Inc., 2011) by relying on heuristics such as file name extensions, shebang lines in executable scripts (e.g., #!/bin/bash), editor mode lines (e.g., -*- mode: python -*-), and *a priori* knowledge about programming language grammars (e.g., their keywords or comment delimiters). More recently PLI methods based on supervised machine learning (ML) have emerged, replacing the need of maintaining complicated heuristics as languages evolve with neural network training.

Depending on the use case, two major classes of ML approaches to PLI have been used (Kiyak et al., 2020): text-based and image-based programming language identification. In text-based approaches source code is represented as character sequences, such as files stored in version control system (VCS) repositories. Image-based approaches can classify raster images showing code, such as screenshots of development environments or individual frames extracted from video programming tutorials.

Image-based PLI models are currently capable of recognizing a limited amount of different programming languages, with a maximum “diversity” of 10 languages found in the literature (Kiyak et al., 2020; Hong, Mizuno & Kondo, 2019). Arguably, recognizing a handful of programming languages could be approached with simple heuristics without incurring the maintenance overhead of (re-)training machine learning models. Hundreds of programming languages exist in the wild, sometimes exhibiting only subtle syntactic differences, and they evolve over time, slowly but regularly (Sammet, 1972). It is at such scale of diversity that PLI approaches based on machine learning would be most useful, but it remains to be seen if it is possible to visually recognize that many programming languages with high accuracy. The first research question we address in this paper is thus:

• **RQ1:** Is it possible to automatically identify the programming language used in code snippet images, among *many languages*, without any *a priori* knowledge about the languages, with high precision and recall performances?

where with “many” we mean an amount comparable to the language diversity supported by practical state-of-the-art PLI tools (machine learning-based or otherwise), in the order of hundreds.

Also, it is not yet established in the literature *what* allows image-based ML models to visually recognize programming languages, especially at this scale of language diversity. Such knowledge would allow in the future to specialize recognition networks and improve performances. The second research question that we address is then:

• **RQ2:** What makes code visually recognizable? Specifically, which classes of characters occurring in code snippets contribute the most to the identification of the programming languages they are written in?

### Paper contributions

We propose an image-based approach for programming language identification that relies on convolutional neural networks (CNNs) which have been pretrained on generic images and subsequently adapted to PLI using transfer learning. We validate the approach on a real-world dataset of 20 million source code files retrieved from popular GitHub repositories, from which we extract a balanced dataset of 300,000 code snippet images encompassing 149 programming languages. We test the approach using three classifiers, each based on a different pretrained CNN for image recognition: a residual network(ResNet) (He et al., 2016), MobileNetv2 (Howard et al., 2017), and AlexNet (Krizhevsky, Sutskever & Hinton, 2017). We positively answer RQ1, by achieving both precision and recall of 92–93% with the ResNet- and MobileNet-based classifiers.

We answer RQ2 by randomly scrambling different character classes (alphabetic characters, decimal digits, symbols) and comparing PLI efficacy. We show that symbols contribute the most to visual recognition of code (halving precision when scrambled), followed by far by alphabetic characters, with decimal digits and indentation having a negligible impact on PLI accuracy.

### Paper structure

We discuss related work and compare it with our contributions in ‘Related Work’. Approach and experimental methodology are described in ‘Methodology’. Experimental results are presented in ‘Results’ and discussed in ‘Discussion’. We discuss threats to validity in ‘Threats to Validity’. ‘Conclusion’ concludes the paper and outlines future work. A complete replication package for this paper is referenced in the “Data Availability” section at the end of the paper.

## Related Work

### Image-based PLI

Kiyak et al. (2020) compared several image- and text-based approaches to Programming Language Identification (PLI). At a glance, Table 1 in their work reports that the maximum diversity supported by image-based PLI among surveyed works was 8 languages, reached by the same authors in Kiyak et al. (2020) with an accuracy of 93.5% on a dataset of 40 K files. We achieve comparable performances (92% precision and recall) with much higher language diversity (149 languages) and on a larger dataset (300 K snippets). Both approaches use Convolutional Neural Networks (CNNs), the main difference being our usage of transfer learning to adapt pretrained image CNNs. In light of the obtained performances, the saving in training effort enabled by transfer learning appears to validate our choice.

Image-based PLI has been attempted by others too. Ott et al. have shown how to use CNNs to identify video frames that contain Java code within video programming tutorials (Ott et al., 2018a) (versus frames not showing code at all) and to distinguish frames containing Java from frames containing Python (Ott et al., 2018b). In the present work we consider a much larger set of languages. They use real images from screencasts and labelling performed manually by students, whereas we use synthetic images and rely on Linguist (GitHub, Inc., 2011) as source of truth.

Hong, Mizuno & Kondo (2019) (not considered in Kiyak et al. (2020)) performed image-based PLI over 10 languages with 90% accuracy, using snippets from StackOverflow and GitHub, rendering them to bitmaps like we do, but using GuessLang (SOMDA, 2017) as source of truth. They use ResNet as a pretrained CNN, which we also considered in this work. In comparison, we achieve a slightly better accuracy at much higher language diversity and we provide a more complete overview of the possible approaches by comparing the results of several CNNs.

To the best of our knowledge no previous work has investigated which visual features contribute to the visual recognition of code snippets, which we address in this paper answering RQ2.

### Other visual artifacts

Visual development artifacts have also been analyzed for uses cases other than PLI. CodeTube (Ponzanelli et al., 2016) uses Optical Character Recognition (OCR) techniques to index the parts of video programming tutorials that contain code fragments and allows to query them as text. Yadid & Yahav (2016) used similar techniques to extract code from video tutorials, joining together snippets that spawn multiple frames with OCR error correction. Zhao et al. (2019) used CNNs to automatically identify common development workflow actions in programming screencasts. The images in our dataset are not from screencasts, but given the high-quality of screencast frames we expect the proposed classifiers to be applicable in that context as well.

### Text-based PLI

Although less relevant for comparison with this paper in terms of approach and field of use, *text*-based PLI has been studied extensively, achieving both good accuracy and high language diversity. Various approaches have been used in the literature: support vector machines(SVM) (Ugurel, Krovetz & Giles, 2002), long short-term memory(LSTM) (Reyes, Ramírez & Paciello, 2016), custom heuristics (Klein, Murray & Weber, 2011), CNNs (Gilda, 2017), and more recently fully-connected neural network classifiers (Del Bonifro, Gabbrielli & Zacchiroli, 2021). The accuracy achieved by text-based PLI has been as high as 97% (in Van Dam & Zaytsev (2016) for 19 languages); language diversity as high as 130 languages (in Del Bonifro, Gabbrielli & Zacchiroli (2021) with 85% accuracy). With the present work we achieve for image-based PLI a language diversity comparable to the maximum one supported by image-based PLI approaches and also higher accuracy.

Heuristic-based programming language recognition tools like sloccount (Wheeler, 2001) and cloc (Danial, 2006) have been available for a long time. Linguist (GitHub, Inc., 2011) is a state-of-the-art PLI tool developed by GitHub, with a self-reported (Ganesan & Foti, 2019) accuracy of 85%. Several PLI studies rely on Linguist as ground truth, as we do in this work. In terms of language diversity, Linguist supports 573 languages, but it requires file extensions—without which performance drops crucially (Ganesan & Foti, 2019) to an F1 score <0.05—which are not always. In particular, file extensions cannot be relied upon when trying to identify the programming language of code snippets and in all other situations in which only limited code portions are available.

## Methodology

In this section we describe the used experimental methodology, encompassing: the starting dataset, code snippets rendering, the classifier architectures and their training, and selective character scrambling.

### Dataset

The experimental dataset consists of a large set of code snippets images obtained by rendering real-world code, an approach shared with other image-based PLI works in the literature. We started from a dataset released by the Software Heritage project (Di Cosmo & Zacchiroli, 2017), consisting of all versions of all source code files collected from the 1000 most popular GitHub repositories on 2019-10-08 (https://annex.softwareheritage.org/public/dataset/content-samples/2019-10-08-github-top1k/). This dataset contains both the actual files (≈ 25 million distinct files) and the associated filenames. We used file extensions as a first indication about the file types (that it is going to be refined later). Initially the dataset contained ≈ 6000 different extensions, from which we excluded all uncommon ones by filtering out those that occur with a frequency <10^−5^ within the dataset, thus obtaining 717 remaining extensions.

As the main goal of this work is the recognition of programming languages, we could not directly use file extensions as labels for supervised machine learning. Hence, we run Linguist (GitHub, Inc., 2011) (a popular choice for source of truth of PLI works in the literature) on all source code files having one of the remaining extensions, while excluding all the files for which Linguist was unable to predict the language. At the end of this step 212 languages remained in the dataset, together with the associated source code files. From this point on classification labels are programming languages (as detected by Linguist) and no longer file extensions.

### Code snippets

The amount of both files and source lines of code (SLOCs) in the dataset at this point were highly unbalanced across languages. For example, popular programming languages such as Python or JavaScript occurs in thousands of source code file examples, whereas other languages only had 100 examples or less. As unbalanced datasets are a well-known issue for supervised machine learning we wanted to mitigate this issue, while at the same time avoiding both high file repetition rate (oversampling) and the exclusion of too many languages (downsampling). Avoiding downsampling is particularly relevant here, since our goal is to assess the feasibility of high-accuracy image-based PLI with *many* languages.

**Figure 1:**
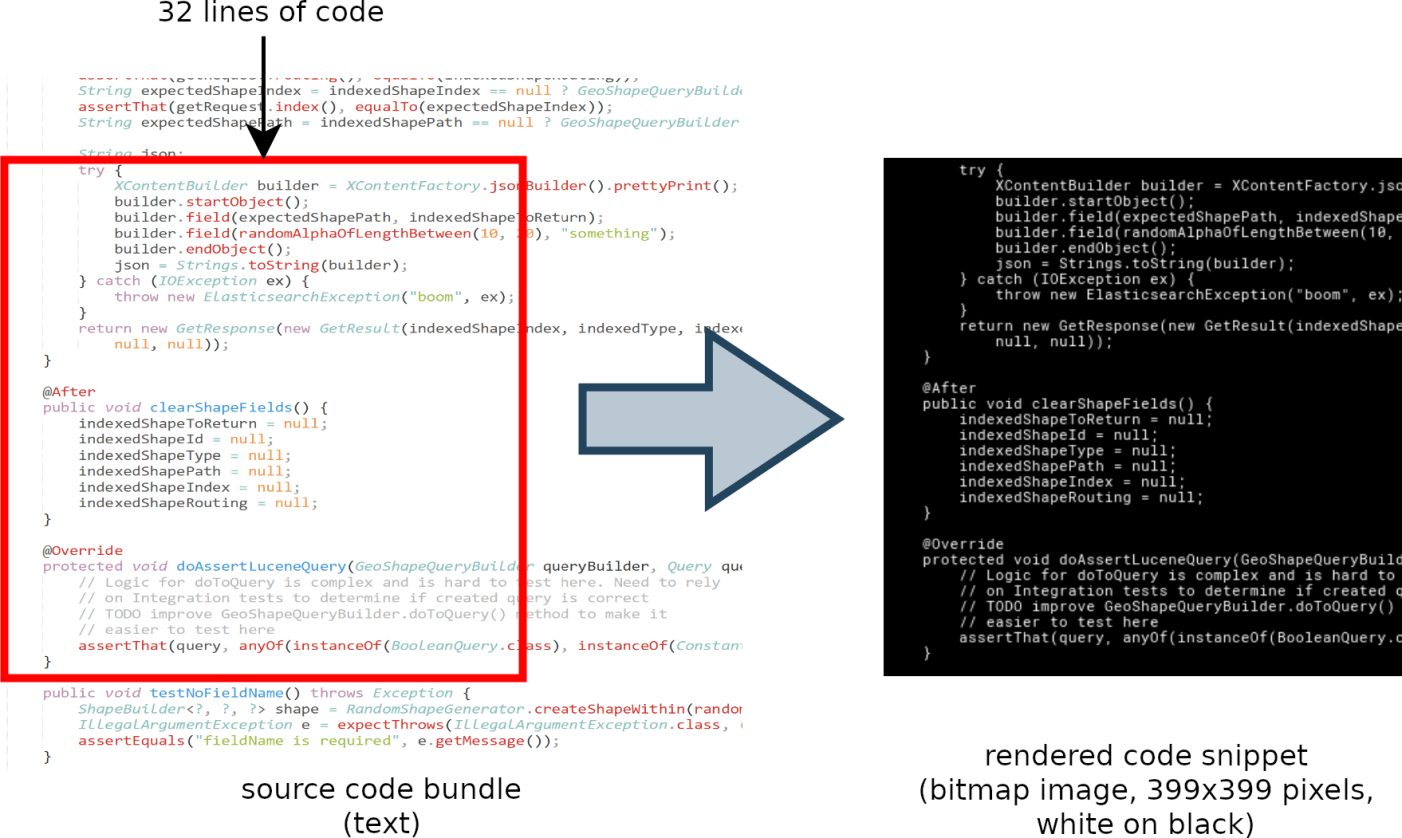
Source lines of code extraction from source code bundles and their rendering.

We exploited the fact that we need code *snippets* images rather than entire source code *files*. As a first step, we created one source code *bundle* for each programming language concatenating together up to 1000 files randomly selected among all the files written in that language. Then we moved a sliding window of 32 SLOCs (see Fig. 1) randomly moves along the vertical axis of each bundle, extracting 2000 code snippets for each language. We took care of ensuring that the snippets belonging to the test set do not overlap SLOCs occurring in the train set. Bundles that did not contain enough SLOCs to allow the extraction of non-overlapping snippets for the test set have been excluded, thus obtaining a total of 149 recognizable languages and 149∗2000 = 298000 labeled snippets.

Finally we rendered each snippet to a raster PNG image of size 399 × 399 pixels by using white on black text typeset in the Roboto Mono monospace font (https://fonts.google.com/specimen/Roboto+Mono) with a font size of 11 points. Note that all obtained images are squared and have the same size, so trimming of long lines could happen, as shown in Fig. 1.

### Classifiers

Convolutional Neural Networks (CNNs) (O’Shea & Nash, 2015) represent the state-of-the-art and the most used neural network architectures for image recognition. CNNs are commonly used for image-based PLI and our approach rely on them as well, with two notable differences from related works in the literature: a large number of classes to be recognized and the use of transfer learning.

Transfer learning (Zhuang et al., 2021) is a well-known machine learning approach that, rather than training models from scratch for a specific classification task, starts from a model that has been pre-trained on a related domain and then adapts it to the target domain. The key advantage of transfer learning is that it allows to obtain good classification performances while using a reduced training set and therefore reduced training costs. Whereas we did have enough data in our dataset to start from scratch, training cost remains an important concern in PLI because, due to the rapid evolution of source code artifacts in the target domain, one has to add to the *initial* training cost that of *periodic* retraining. This domain-specific aspect of the problem led us to the decision of using transfer learning.

**Figure 2:**
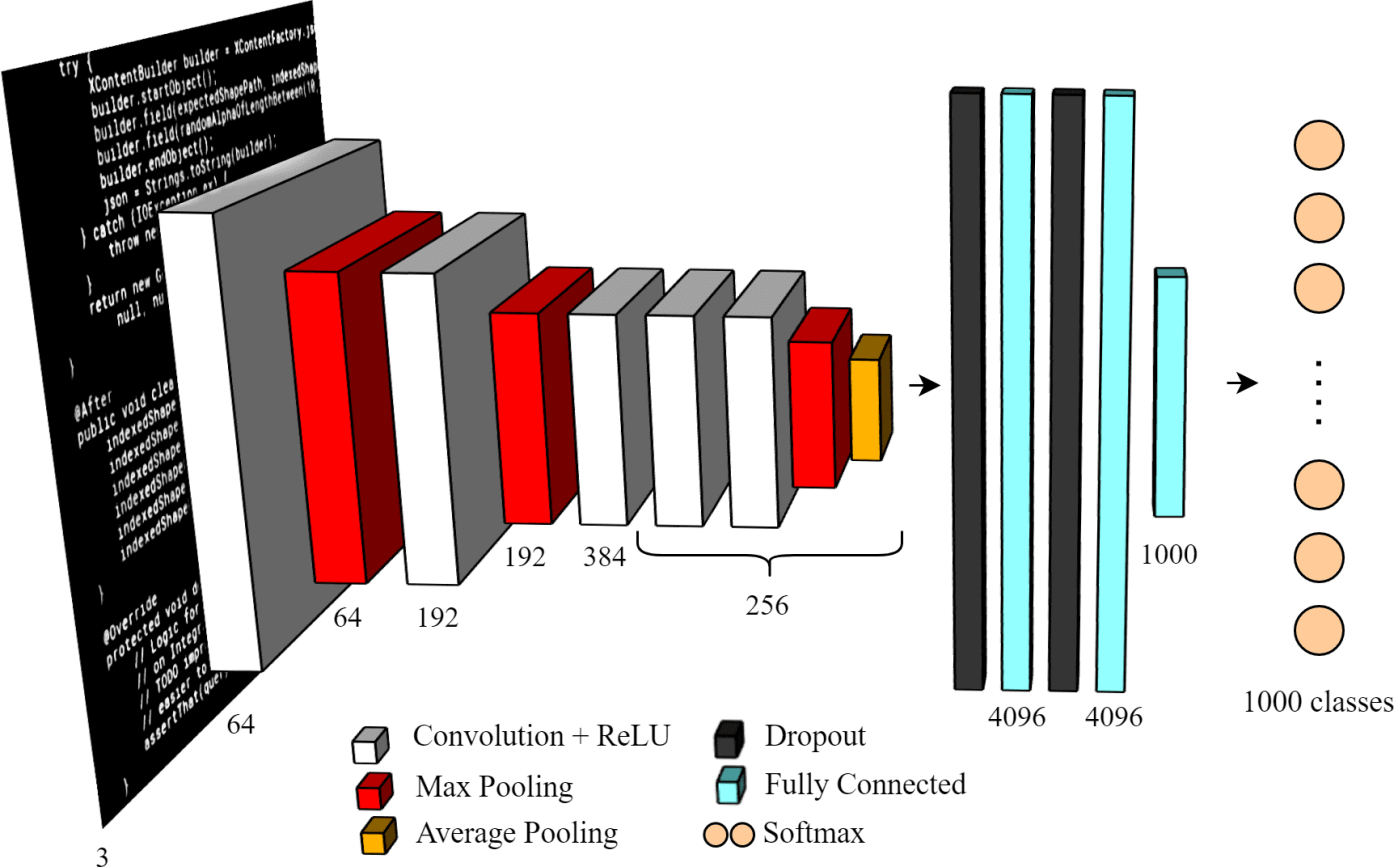
AlexNet architecture, the simplest pre-trained CNN among those we specialized for visual code recognition.

**Figure 3:**
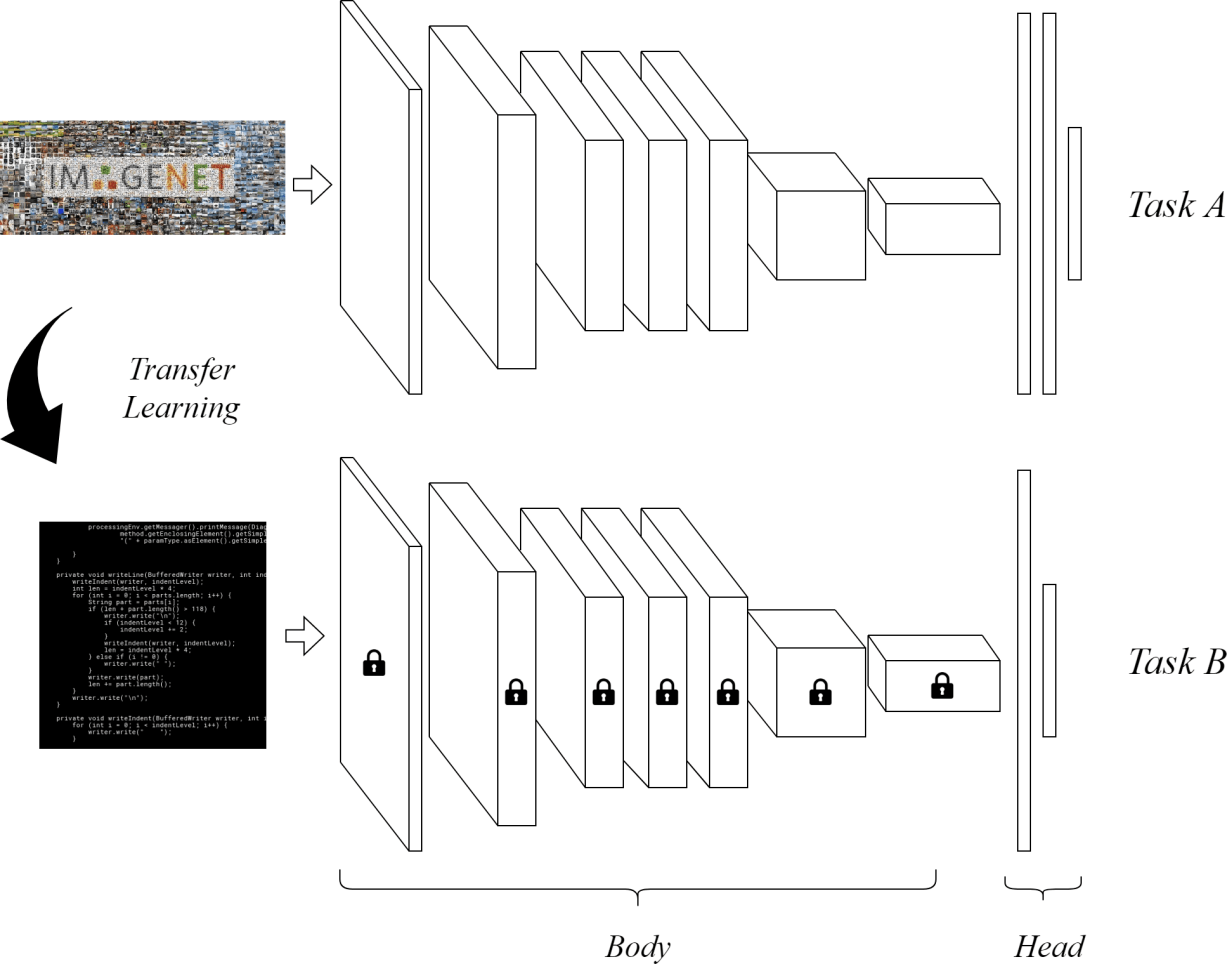
Using transfer learning to adapt CNNs trained on ImageNet to visually recognize code snippets.

We compared the performances of three classifiers based on three different CNN architectures pre-trained for images recognition: ResNet34 (He et al., 2016), MobileNetv2 (Howard et al., 2017) and AlexNet (Krizhevsky, Sutskever & Hinton, 2017). The first two have about 30 layers each, while the latter has 8 layers. We show in Fig. 2 the architecture of AlexNet, as it is the simplest to fully depict; the other two architecture are similar but significantly deeper. The three CNNs we used were all pre-trained on ImageNet (Deng et al., 2009), a generic image dataset composed of more than 14 million images classified into 20000 classes. This allowed us to benefit from the features and invariants learned on ImageNet in order to perform image-based PLI, as shown in Fig. 3.

### Training

As a preliminary step in the model training we replaced the classification layer (or *head*) of each CNN—initially composed of 1000 output neurons, as required by the ImageNet classification task—with a head composed of 149 output neurons, corresponding to the cardinality of our set of programming languages to recognize. We applied a (usual) 80/20% split to our dataset twice to obtain the training, validation, and test sets. First we kept aside 20% of the obtained code bundles for testing and then further split the rest to obtain the training and validation sets. This resulted in an overall partition of all code bundles in three sets: 64% for training, 16% for validation, and 20% for testing.

We then applied a two-step training procedure to all three CNN architecture. As a first step the weights of the CNN (the *body*) have been frozen so that training could only affect the substituted head. This way the features previously learned by the convolutional layers during ImageNet training are exploited to make predictions about the new classification task (Fig. 3).

After a few epochs of training we moved to the second training step, where all the weights are unfrozen, thus allowing training updates all over the architecture. A slightly lower learning rate is used in the second training step with respect to the first, so that the network can adapt to the task of image-based PLI without completely forgetting what the network has learned about images in general.

Table 1 shows the total training times for the three architectures, as well as a breakdown per training step, the number of training epochs, and the average per-epoch training time in each case. Training has been performed on a Linux machine equipped with an Intel Xeon 8 core 2.1 GHz CPU, Nvidia Titan XP GPU and 96 GiB of RAM. The slowest architecture is MobileNet, which took around 7 h to complete both training steps; the fastest architecture is AlexNet, requiring less than 2 h of total training time.

**Table 1:** Training times per epoch (minutes and seconds), number of epochs and total training times (hours, minutes and seconds) for AlexNet (A), MobileNet (M) and ResNet (R) -based models for the two considered steps of training.

	**Per-epoch training time (avg.)**			**Epochs**			**Total training time**		
	A	M	R	A	M	R	A	M	R
step 1	6m33s	22m20s	20m04s	8	8	8	52m23s	2h58m39s	2h40m29s
step 2	6m49s	30m32s	26m46s	8	7	7	54m29s	3h44m41s	3h07m20s
total	13m22s	52m52s	46m50s				1h46m52s	6h43m20s	5h47m49s

Improvements in the training process, including finding a good balance between underfitting and overfitting phenomena, can be obtained by setting suitable values for several network hyperparameters. We mainly focused on tuning the learning rate (LR) while the other hyperparameters, such as epochs and batch size, have been kept at fixed values. For LR tuning we used the One Cycle Policy (Smith, 2017), where the LR cyclically varies within a certain range allowing improvements both in classification accuracy and training time. The LR range’s upper bound has been determined during a pretraining phase according to a method recently proposed by Smith (Smith, 2018), which represents an efficient alternative to the common random search technique. We run one epoch of training starting with a small LR and gradually increasing it at each training iteration, while recording the validation loss values. At the beginning the loss decreases, then reaches its minimum, and then starts to increase: such a minimum indicates the LR value that we have retained. The lower bound of the LR range was set to be $ \frac{1}{25} $ of the upper bound.

### Scrambling

When using CNNs for image-based PLI, learned features are automatically extracted by the network during training. In order to better understand what are the domain features (indentation, particular character classes, text placement, etc.) that allow the networks to visually recognize programming languages we selectively added noise to the code snippet images of the test set. This allowed us to determine which characters impact the most language identification capabilities, answering RQ2.

**Figure 4:**
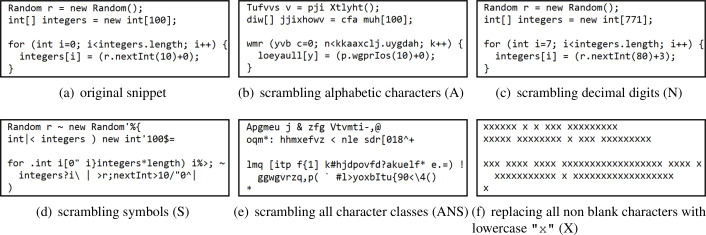
Java code snippet in original form v. several scrambled variants. (A) original snippet. (B) scrambling alphabetic characters (A) (C) scrambling decimal digits (N) (D) scrambling symbols (S) (E) scrambling all character classes (ANS) (F) replacing all non-blank characters with lowercase ”x” (X).

We defined three classes of characters that are commonly used to define lexemes in the syntax of programming languages: *alphabetic* characters (denoted by A), *numeric* decimal digits (N), and *symbols* (S) for the remaining non-blank characters (mostly punctuation characters, mathematical symbols, and parentheses). Scrambling consists in replacing each character of a class being scrambled by another character, randomly selected within the same class, while preserving string lengths. Figure 4 shows some examples of scrambling results.

We first applied scrambling of one character class at a time, without changing characters belonging to other classes, obtaining 3 scrambled test sets denoted A, N, and S. We then scrambled pairs of character classes together, obtaining 3 additional scrambled test sets denoted AN, AS, and NS; then we scrambled all the three character classes together, obtaining the scrambled test set denoted ANS. Finally we considered the extreme case in which every characters except blanks have been replaced by a (lowercase) x character, preserving only the overall code “layout”, as dictated by code indentation, obtaining the scrambled test set denoted by (uppercase) X.

## Results

We implemented in Python the three classifiers described in the previous section, using the PyTorch framework (Paszke et al., 2019). The three implementations are available as part of the complete replication package for this paper (see ‘Conclusion’).

For each architecture the training phase has been performed on the original non-scrambled dataset and then repeated for each classifier and for each scrambled version of the dataset. In order to be able to compare results among the different architectures we used a fixed PRNG (pseudorandom number generator) seed to make sure that images were processed in the same order during both training and evaluation.

### Image-based PLI performances with many languages

On the non-scrambled dataset, after 8 epochs of the first training phase—in which only the weights of the classifier’s head were able to be updated—the ResNet- and MobileNet-based classifiers reached ≈90% accuracy on the validation set, while the AlexNet-based model reached only ≈60%. Performances improved for all models after the fine-tuning phase—when all weights could be updated, although at a lower learning rate. After 7 epochs of fine-tuning ResNet accuracy reached ≈92% (+2%), MobileNet ≈93% (+3%), and AlexNet ≈84% (a significant +24%, but still the worst performing classifier overall).

Several performance measures have been computed to evaluate the performance of the three classifiers: precision (P), recall (R) and F1 measure (F1). Since PLI classification is a single-label, multi-class problem, P, R and F1 are computed on the confusion matrix obtained by applying the trained models to the test set separately for each class, as follows:

(1) \begin{eqnarray*}{P}_{i}= \frac{{C}_{ii}}{\sum _{j=0}^{149}{C}_{ji}} = \frac{T{P}_{i}}{T{P}_{i}+F{P}_{i}} \end{eqnarray*}

(2) \begin{eqnarray*}{R}_{i}= \frac{{C}_{ii}}{\sum _{j=0}^{149}{C}_{ij}} = \frac{T{P}_{i}}{T{P}_{i}+F{N}_{i}} \end{eqnarray*}

(3) \begin{eqnarray*}F{1}_{i}= \frac{2{P}_{i}{R}_{i}}{{P}_{i}+{R}_{i}} \end{eqnarray*}

where: the generic element *C*_*i*,*j*_ of the confusion matrix represents the amount of samples of the *i*th class that the model classified as *j*th class instances; *TP*_*i*_ is the amount of true positives (i.e., instances of the *i*th class that have been correctly classified); *FP*_*i*_ is the amount of false positives (instances that were wrongly classified as belonging to the *i* − *th* class); and *FN*_*i*_ is the amount of false negatives (*i*th class instances that were not classified as such).

The micro and macro averages for the three metrics P, R and F are reported in Table 2, as a summary of the overall performances of each classifier. Micro average is obtained by considering the total of the true/false positives/negatives among all classes obtained from the confusion matrix and by applying the measures formulae. Macro average is obtained by summing up the *P*_*i*_, *R*_*i*_ or *F*1_*i*_ values obtained for each class and dividing by the number of classes.

**Table 2:** Recognition performances for three pretrained CNNs, fine-tuned for image-based programming language identification.

	ResNet34			MobileNetv2			AlexNet		

	**P**	**R**	**F1**	**P**	**R**	**F1**	**P**	**R**	**F1**
**Micro avg.**	0.92	0.92	0.92	0.92	0.92	0.92	0.83	0.83	0.83
**Macro avg.**	0.92	0.92	0.92	0.93	0.92	0.92	0.83	0.83	0.83

Two aspects are worth noticing: first, performances range from acceptable to very good for all classifiers, with precision in the 83–93% range (depending on the base CNN) and recall in the 83–92% range. Second, the ResNet- and MobileNet-based classifiers significantly outperform the AlexNet-based one, to better generalize to the PLI dataset within a limited number of training epochs. Performances of the ResNet- and MobileNet-classifiers are almost as good (−1.5%) as the state-of-the-art in image-based PLI, in spite of a ×15-time increase in language diversity and of reduced training costs.

A detailed breakdown of performance results for each language is given in Appendix A. We notice from it that most languages perform very well, close to the overview given by the aggregate performance metrics. Most of the languages that perform poorly still perform well above 80% precision with the best performing classifiers. The languages that perform the worst tend to have common syntactic characteristics either among them or with other languages included in the dataset. This is the case when a language is a subset of another one, as it is for example for Objective-C and Objective-C++; and yet the two languages are recognizable with 76–82% precision by the MobileNet-based classifier. Other low-precision cases are related to languages that can embed other languages, such as HTML, JavaScript, JSX, Less, and XSLT. All classifiers exhibit weaknesses in visually recognizing these languages.

### What makes code snippets visually recognizable?

The three classifiers trained on the original trainset have been then tested on the scrambled versions too. Performance results are presented in Table 3 for each architecture on the various dataset versions. The results provides some insights on what makes a code snippet visually recognisable, as we discuss below.

**Table 3:** PLI performances with and without scrambling. From left to right: no scrambling (Orig), scrambling of alphabetic characters (A), digits (N), symbols (S), combinations of them (AN, AS, NS, ANS) and substitution of all non-blank characters for x (X).

	Orig	A	N	S	AN	AS	NS	ANS	X
	ResNet34								
**Precision**	0.92	0.87	0.92	0.47	0.87	0.34	0.48	0.35	0.01
**Recall**	0.92	0.85	0.92	0.33	0.85	0.20	0.33	0.19	0.17
**F1**	0.92	0.86	0.92	0.39	0.86	0.25	0.39	0.25	0.01
	MobileNetv2								
**Precision**	0.92	0.89	0.93	0.42	0.89	0.31	0.44	0.29	0.04
**Recall**	0.92	0.88	0.92	0.25	0.88	0.16	0.25	0.16	0.05
**F1**	0.92	0.88	0.93	0.31	0.89	0.21	0.32	0.21	0.04
	AlexNet								
**Precision**	0.83	0.75	0.83	0.57	0.75	0.44	0.58	0.44	0.09
**Recall**	0.83	0.72	0.83	0.49	0.72	0.31	0.50	0.31	0.05
**F1**	0.83	0.73	0.83	0.53	0.73	0.37	0.53	0.36	0.06

We can see that randomly scrambling decimal digits alone (dataset “N”) induces no degradation in PLI performances for the three classifiers. Scrambling alphabetic characters alone (dataset “A”) has a mild performance impact, degrading precision and recall by 3–11%, depending on the CNN, with AlexNet being the most affected one. Scrambling symbol characters alone (punctuation, operators, parentheses, etc; dataset “S”) on the other hand is enough to have a dramatic effect on the performances of every considered architecture, inducing an impressive drop in both precision and recall in the 2–4 × range. This degradation is likely due to the syntactic (and hence visual) importance that punctuation characters play in programming languages and the highly different usage of them across different languages.

Scrambling several character classes at once (datasets: “AN”, “AS”, “NS”, and “ANS”) appears to simply combine the effects of scrambling individual character classes. AN still performs relatively well (because symbols are *not* scrambled), all the datasets which *also* involve symbol scrambling perform badly, and scrambling all three character classes at once exhibits the worst performances.

Performances for the “X” dataset, where all non-blank characters have been replaced by x, are below 10% for both precision and recall in most cases, reaching as low as 1% for the precision of the ResNet-based classifier. It appears that the code “layout” alone, as captured by indentation, is nowhere near enough to make programming languages visually recognizable.

## Discussion

Based on the obtained results we can now affirmatively answer RQ1: it is possible to automatically recognize up to 149 different programming languages in brief code snippet images, with high accuracy. This is a significant step forward in the state-of-the-art of image-based PLI, which was limited to recognize up to 10 languages. This result paves the way to use of image-based PLI in real-world settings, where much more than a handful of programming languages need to be handled. It is worth noting that we stopped at 149 languages only to avoid dataset imbalance, not due to intrinsic limitations in the proposed approach. Using larger code bases (Abramatic, Di Cosmo & Zacchiroli, 2018; Ma et al., 2019) as training datasets it should be possible to achieve even higher language diversity without significant reductions in identification accuracy.

Regarding RQ2, we have gathered evidence that symbols—punctuation, arithmetic operators, parentheses, etc.—are the characters that impact the ability to visually recognize programming languages the most, making precision halve and recall diminish by $ \frac{2}{3} $ when scrambled and with the best-performing classifier. Alphabetic characters have a minor impact (a few percentage points drop), whereas decimal digits have no measurable impact. The difference among classes makes intuitive sense, but the impact of symbol scrambling remains remarkable, especially considering how symbols tend to be reused for similar needs across different languages (e.g., many languages share the use of arithmetic operators or, to a lesser extent, of ”;”). A more fine grained analysis of which *individual* symbols impact recognition the most is needed as future work.

We intuitively expected the “layout” of the code alone, as captured by indentation, to perform better than what we have observed with the “all x” scrambled dataset, which performed terribly with all classifiers in our experiments. In particular, we expected indentation to be a tell for at least the languages where indentation is syntactically meaningful, such as Python. That factor is probably contrasted by the fact that proper indentation is a coding best practice for all languages, and certainly so in a dataset assembled from *popular* open source projects. This could make good indentation a common trait of all languages and snippets, from which nothing can be learned by a trained classifier.

**Figure 5:**
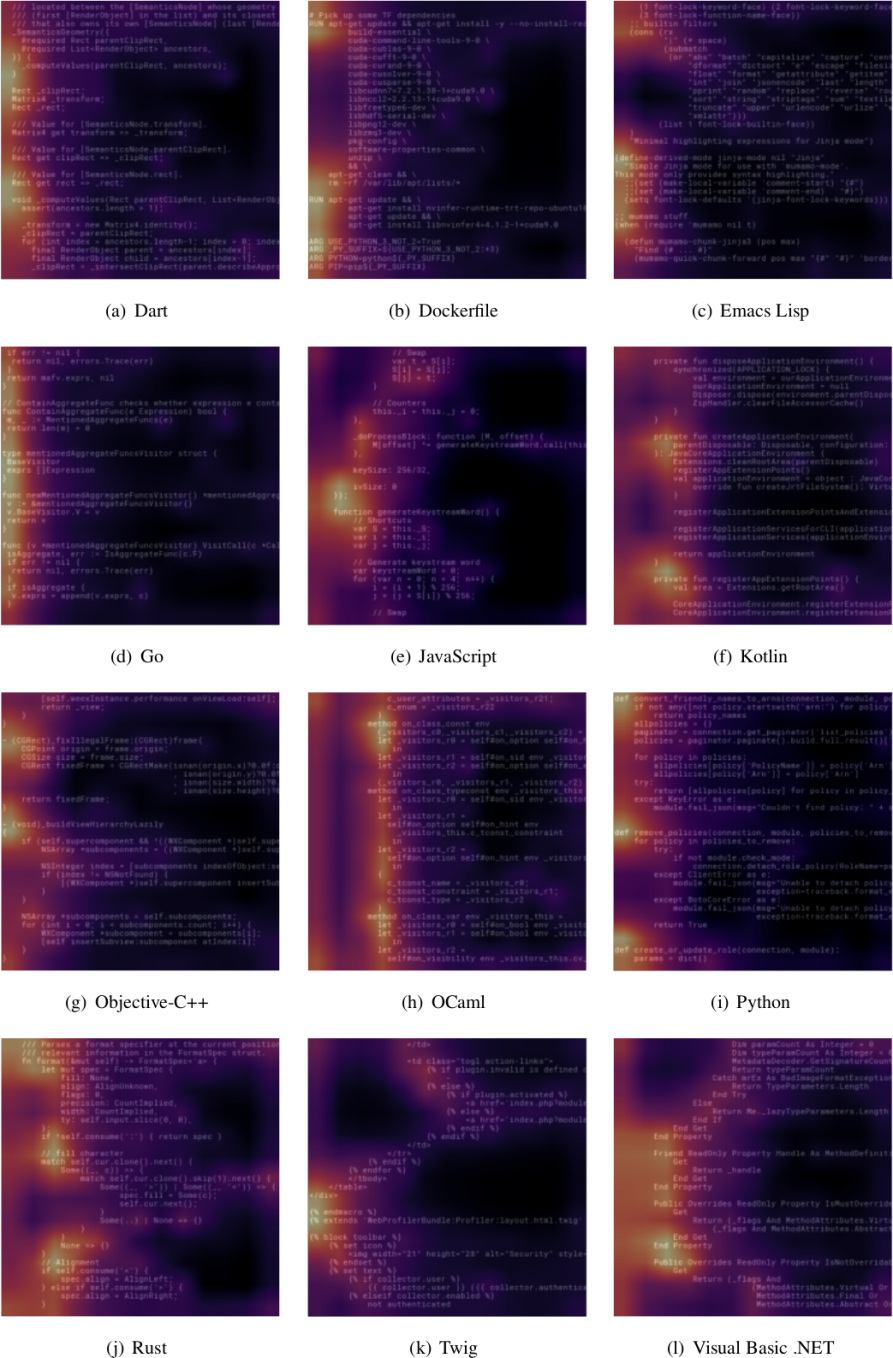
Class Activation Map (CAM) heatmaps for selected code snippets in various languages, for the ResNet-based classifier. (A) Dart. (B) Dockerfile. (C) Emacs Lisp. (D) Go. (E) JavaScript. (F) Kotlin. (G) Objective-C++. (H) OCaml. (I) Python. (J) Rust. (K) Twig. (L) Visual Basic NET.

To get a *qualitative* feeling of where the classifiers gather the most relevant information for image-based PLI, we show in Fig. 5 the class activation map (CAM) (Zhou et al., 2015) heatmaps for selected snippets. We have generated CAMs from the ResNet-based classifier, using PyTorch hooks just after the last convolutional layer. Colors in CAMs highlight which image parts contributed the most to the final classifier decision, helping with the understandability of machine learning models.^1^ 1These maps can be seen as heatmaps, with black/blue colors indicating low temperatures and the scale going up to yellow for high temperature in the usual way. High temperature in our case means high contribution to the final classifier decision.

A few observations about these CAMs are in order. First, CAMs highlight the fact that the *beginning* of code lines is very relevant for classifier decisions. This relates to the importance of symbol characters—which are often found at the beginning of each line, like parentheses, and are also highlighted by CAMs elsewhere in code snippets—but it appears to go deeper than that. For instance it seems that for several languages the CNN has learned to recognize full language keywords, such as ”def” for the Python programming language and other keywords for Dockerfiles and Visual Basic. Second, CAMs confirm that indentation is not useful for PLI: spaces at the beginning of code lines remain almost invariably in the dark. These are just some preliminary considerations based on CAMs, whose exhaustive analysis in the context of image-based PLI was out of scope for this paper, but constitutes a promising lead for future work.

In terms of machine learning architectures we have shown that transfer learning starting from pretrained image CNNs is a viable option for image-based PLI, an approach that had received little attention in the literature for this domain thus far. With respect to starting from scratch, our approach offers the benefits of cheaper (re)training, reducing maintenance costs. Considering the very marginal reduction in precision in comparison to previous work (≤1.5% with respect to Kiyak et al. (2020)), which is probably in large part imputable to the much higher language diversity in our experiments, the pros/cons balance seems to tilt towards pretrained CNNs and transfer learning.

In this respect it seems worth to explore side-tuning (Zhang et al., 2020), a recent technique for transfer learning which consists in adapting a pre-trained network by training a lightweight “side” network that is then fused with the (unchanged) pre-trained network via summation. Side-tuning works very well in several scenarios (Zhang et al., 2020) and it has recently been shown (Zingaro, Lisanti & Gabbrielli, 2021) to be applicable to multimodal document classification, where diverse data sources such as text and images are considered, improving document classification accuracy with respect to the state of the art. Such a multimodal approach could be naturally applied to PLI, by interpreting code snippets as both images and text. The empirical validation of the applicability of side-tuning to PLI is left as future work.

## Threats to Validity

We rely on Linguist (GitHub, Inc., 2011) as source of truth for what is the “real” language of a code snippet. Whereas this is a common choice in the PLI literature due to the efficacy, efficiency, and broad language support of Linguist, it still means that our precision and recall results should be diminished by Linguist’s performances, which is reported by GitHub as having 85% accuracy (Ganesan & Foti, 2019). As we are comparing with other works in the literature that also used Linguist as source of truth, this fact does not impact the improvement in language diversity that we introduce with this paper.

We used synthetic images rendered from textual code snippets instead of real-world images. Both approaches can be found in the PLI literature and we compare well with previous works that also used rendered images. Moreover, works that use “real” images rely for the most part on video frames extracted from programming tutorials. Those tutorials are generally produced as screencasts, in which programming editors and IDEs are recorded directly from the desktop environment (rather than, say, from a physical camera pointed at the screen), resulting in very high-quality video frame images. Therefore we do not expect our performances to be significantly impacted by the switch from synthetic images to screencast video frames. Visual recognition of actual real-world images—e.g., pictures of billboards showing code or movie frames of screens showing code—would be a different matter, but it is a challenge we share with most works in the image-based PLI literature.

We relied on Software Heritage as data source (instead of *ad hoc* crawling) and used a dataset corresponding to code retrieved from the most popular GitHub repositories. We further cleaned up all unrecognizable and unpopular languages according to the data pipeline discussed in ‘Methodology’. There is certainly a bias in this process, tilting in favor of “good” snippets rather than considering a wide spectrum of good and bad ones. We believe that our choice in this respect leads to comparable results to other approaches (e.g., retrieving snippets from StackOverflow or extracting video frames), as those solutions are also characterized by a significant selection bias for quality (the barrier for posting a snippet on StackOverflow or publishing a video tutorial being much higher than that of pushing code to GitHub).

In terms of dataset size, and according to a recent literature review (Kiyak et al., 2020), our experiments have been conducted over 12–15 × more snippets than the largest empirical studies in *image*-based PLI. Rather, our dataset size is inline with the largest datasets used for *text*-based PLI. It is worth noting that we have arguably amplified the number of snippets that form our experimental dataset, in the sense that we have extracted several snippets from each source code file. At the same time we have been careful in not extracting overlapping snippets for the training set, mitigating (if not fully neutralizing) this threat.

## Conclusion

As a first contribution we have shown that image-based programming language identification (PLI) can be performed with high precision and recall even when many different programming languages (149 in our experiments) are considered. This corresponds to a 15 × increase in language diversity with respect to the state-of-the-art, incurring no significant loss (≤1.5%) in accuracy. We established this result on a dataset consisting of ≈ 300,000 code snippets extracted from 1,000 popular GitHub repositories. We have used three classifiers based on convolutional neural networks (CNNs) pretrained for images (ResNet34, MobileNetv2, and AlexNet) then adapted to the PLI domain using transfer learning, with significant reductions in terms of training cost with respect to the common approach in the PLI literature of training from scratch. The ResNet-based classifier performed best, achieving 92% in both precision and recall.

Our second contribution is a quantitative exploration of what makes programming languages visually recognizable. By selectively scrambling different character classes in the original dataset we found evidence that symbolic characters such as punctuation, arithmetic operators, and parentheses contribute the most to visual programming language recognition, followed (by far) by alphabetic characters, with no to negligible impact by decimal digits and indentation in our experiments. A preliminary qualitative exploration of class activation maps(CAMs) supports these findings and also suggests that CNN-based classifiers for code snippet images might be able to autonomously learn to spot language keywords, without any *a priori* knowledge of programming language grammars.

### Future work

Several leads remain to be explored as future work. The introduced classifiers should be empirically validated on non-synthetic images extracted from programming tutorial videos and pictures containing code, e.g., billboards or pictures of screens showing code. The former should be straightforward, the latter more challenging and also a significant departure from the tradition of image-based PLI.

On the front of synthetic images, where by construction both textual and visual representations of code snippets are available, we intend to explore side-tuning as part of classifier training, to verify if it can further improve PLI performances, as it did in other problem spaces.

With the analysis of scrambled character classes we have only scratched the surface of the understanding of what makes programming languages visually identifiable. Fine-grained analyses are needed to identify which code parts matter the most for CNN-based classifiers. CAM-based observations seem to hint at the importance of keywords, which we intend to explore more, together with finer-grained scrambling and spatial layout analyses. If a clear understanding of what makes code visually recognizable could be reached, it is possible that dedicated feature engineering could lead to even more accurate image-based PLI classifiers.

Finally, even though we have been able to visually recognize almost 150 programming languages, we intend to try pushing further this limit and reach hundreds of recognized languages, retaining high accuracy. The main difficulty here is the lack of a suitably balanced dataset, a task that we intend to address in the near future by perusing large source code archives.

##  Supplemental Information

10.7717/peerj-cs.631/supp-1 Supplemental Information 1 Replication package

## Appendix

### Per-language results

The following table reports the disaggregated identification performance (P, R and F1-score) for each programming language and for the three classifiers described in ‘Methodology’, on the original (non-scrambled) dataset.

**Table A1:**

Language	ResNet34			MobileNetv2			Al exNet		
	**P**	**R**	**F1**	**P**	**R**	**F1**	**P**	**R**	**F1**
ANTLR	0.95	0.99	0.97	0.97	0.99	0.98	0.94	0.97	0.96
ActionScript	0.78	0.91	0.84	0.76	0.93	0.83	0.65	0.72	0.68
AGC	0.99	1.00	1.00	0.99	1.00	0.99	0.97	0.99	0.98
AsciiDoc	0.98	0.94	0.96	0.98	0.95	0.97	0.78	0.86	0.81
Assembly	0.94	0.87	0.90	0.97	0.88	0.92	0.86	0.83	0.84
Batchfile	0.98	0.99	0.98	1.00	0.99	0.99	0.91	0.94	0.93
C	0.84	0.92	0.88	0.83	0.94	0.88	0.73	0.75	0.74
C#	0.83	0.91	0.87	0.88	0.91	0.89	0.74	0.83	0.78
C++	0.64	0.66	0.65	0.69	0.70	0.70	0.47	0.39	0.43
CMake	0.97	0.98	0.98	0.97	0.99	0.98	0.81	0.89	0.84
CSON	1.00	0.99	1.00	1.00	0.99	1.00	0.96	0.99	0.98
CSS	0.81	0.87	0.84	0.78	0.91	0.84	0.68	0.72	0.70
CSV	0.97	0.58	0.73	0.98	0.57	0.72	0.93	0.40	0.56
Cabal Config	1.00	0.99	0.99	0.99	1.00	0.99	0.98	0.99	0.99
Cap’n Proto	1.00	1.00	1.00	1.00	1.00	1.00	0.96	0.99	0.98
Clojure	0.98	0.96	0.97	0.96	0.97	0.97	0.84	0.90	0.87
CoffeeScript	0.97	0.88	0.92	0.96	0.89	0.92	0.83	0.85	0.84
Crystal	0.83	0.89	0.86	0.84	0.86	0.85	0.73	0.76	0.74
Cuda	0.78	0.88	0.83	0.72	0.91	0.80	0.56	0.81	0.67
Cython	0.84	0.86	0.85	0.83	0.90	0.87	0.66	0.76	0.70
DIGITAL CL	1.00	0.99	1.00	1.00	0.99	1.00	0.96	0.99	0.98
Dart	0.94	0.95	0.94	0.93	0.96	0.95	0.80	0.84	0.82
Diff	0.97	0.97	0.97	0.99	0.96	0.97	0.93	0.93	0.93
Dockerfile	0.99	1.00	1.00	1.00	1.00	1.00	0.98	0.99	0.99
eC	0.96	0.97	0.97	0.97	0.98	0.98	0.88	0.95	0.91
EJS	0.96	0.88	0.92	0.96	0.92	0.94	0.83	0.81	0.82
EML	0.92	0.97	0.94	0.92	0.96	0.94	0.89	0.85	0.87
Elixir	0.98	0.95	0.96	0.93	0.92	0.93	0.92	0.84	0.88
Emacs Lisp	0.98	0.96	0.97	0.98	0.98	0.98	0.82	0.83	0.82
Erlang	0.98	0.98	0.98	0.98	0.99	0.98	0.94	0.95	0.94
fish	0.98	0.99	0.99	0.99	0.99	0.99	0.93	0.91	0.92
FreeMarker	0.90	0.95	0.93	0.91	0.95	0.93	0.86	0.92	0.89
GAP	0.96	0.89	0.93	0.95	0.91	0.93	0.86	0.77	0.81
GDB	1.00	1.00	1.00	1.00	1.00	1.00	0.98	0.99	0.99
GLSL	0.96	0.94	0.95	0.96	0.92	0.94	0.84	0.81	0.83
GN	0.99	1.00	0.99	1.00	1.00	1.00	0.91	0.99	0.95
Gettext C.	0.98	1.00	0.99	0.98	1.00	0.99	0.99	0.99	0.99
Gherkin	0.98	0.99	0.99	1.00	0.99	1.00	1.00	0.95	0.97
Go	0.92	0.81	0.86	0.93	0.80	0.86	0.70	0.70	0.70
Gradle	0.91	0.93	0.92	0.94	0.93	0.93	0.70	0.74	0.72
GraphQL	0.99	0.98	0.98	0.99	1.00	0.99	0.96	0.90	0.93
Graphviz	0.99	0.96	0.97	1.00	0.97	0.98	0.97	0.92	0.94
Groovy	0.89	0.91	0.90	0.86	0.92	0.89	0.71	0.79	0.75
HAProxy	1.00	0.99	1.00	0.99	1.00	1.00	0.98	0.99	0.98
HTML	0.79	0.54	0.64	0.81	0.60	0.69	0.69	0.53	0.60
HTML+Django	0.89	0.95	0.92	0.75	0.95	0.84	0.83	0.92	0.87
HTML+ERB	0.85	0.89	0.87	0.90	0.90	0.90	0.74	0.80	0.77
HTML+Razor	0.94	0.94	0.94	0.97	0.93	0.95	0.83	0.86	0.85
Hack	0.93	0.95	0.94	0.96	0.96	0.96	0.90	0.92	0.91
Haml	1.00	0.99	1.00	1.00	0.99	1.00	0.97	0.97	0.97
Handlebars	0.97	0.93	0.95	0.96	0.94	0.95	0.91	0.85	0.88
Haskell	0.95	0.70	0.81	0.96	0.72	0.82	0.81	0.64	0.71
INI	0.92	0.99	0.95	0.93	0.99	0.96	0.84	0.92	0.88
Ignore List	1.00	0.99	1.00	0.99	1.00	1.00	0.96	0.99	0.97
Inno Setup	1.00	0.97	0.99	0.99	0.97	0.98	0.98	0.96	0.97
JSON	0.98	0.94	0.96	0.97	0.95	0.96	0.79	0.40	0.53
JSON5	1.00	0.99	1.00	0.99	1.00	0.99	1.00	0.99	0.99
JSX	0.72	0.56	0.63	0.76	0.68	0.72	0.52	0.49	0.51
Java	0.81	0.81	0.81	0.84	0.81	0.83	0.69	0.59	0.64
Java Prop.	0.97	0.99	0.98	0.99	0.98	0.98	0.83	0.93	0.88
Java SP	0.96	0.93	0.95	0.98	0.93	0.96	0.92	0.84	0.88
JavaScript	0.59	0.62	0.60	0.59	0.58	0.59	0.41	0.36	0.38
Jison	0.97	0.99	0.98	0.98	0.99	0.99	0.94	0.99	0.97
Julia	0.91	0.85	0.88	0.88	0.84	0.86	0.76	0.78	0.77
Jupyter Not.	0.97	0.99	0.98	0.97	0.99	0.98	0.90	0.94	0.92
Kotlin	0.91	0.95	0.93	0.92	0.97	0.94	0.69	0.84	0.76
LLVM	1.00	0.84	0.92	0.99	0.88	0.93	0.98	0.68	0.80
Less	0.67	0.68	0.67	0.76	0.67	0.71	0.52	0.46	0.49
Lex	0.95	0.91	0.93	0.98	0.92	0.95	0.88	0.87	0.88
Linux KM	1.00	1.00	1.00	1.00	1.00	1.00	1.00	1.00	1.00
Lua	0.83	0.94	0.88	0.96	0.94	0.95	0.73	0.82	0.77
M4Sugar	0.98	0.94	0.96	1.00	0.93	0.96	0.91	0.83	0.87
MLIR	0.98	0.99	0.98	0.98	0.99	0.99	0.96	0.98	0.97
Makefile	0.88	0.97	0.92	0.88	0.97	0.92	0.77	0.93	0.84
Mako	0.99	0.97	0.98	0.99	0.96	0.97	0.96	0.90	0.93
Markdown	0.85	0.93	0.89	0.91	0.90	0.90	0.72	0.58	0.64
Micr. DSP	1.00	1.00	1.00	1.00	1.00	1.00	1.00	1.00	1.00
MMS	0.99	0.99	0.99	0.99	1.00	1.00	0.97	0.99	0.98
Assembly	0.69	0.71	0.70	0.72	0.72	0.72	0.44	0.53	0.48
NASL	0.93	0.81	0.87	0.91	0.81	0.85	0.89	0.78	0.83
NSIS	0.99	1.00	0.99	0.96	1.00	0.98	0.92	0.96	0.94
OCaml	0.95	0.91	0.93	0.98	0.94	0.96	0.88	0.75	0.81
Object.-C	0.71	0.81	0.75	0.76	0.81	0.78	0.60	0.66	0.63
Object.-C++	0.73	0.62	0.67	0.82	0.71	0.76	0.60	0.49	0.54
Objective-J	0.88	0.93	0.90	0.86	0.93	0.90	0.80	0.88	0.84
OpenCL	0.91	0.96	0.93	0.92	0.97	0.94	0.76	0.90	0.83
OpenType FF	1.00	0.96	0.98	0.99	0.93	0.96	0.97	0.82	0.89
Org	1.00	0.99	1.00	0.99	0.99	0.99	0.93	0.94	0.94
PHP	0.90	0.89	0.89	0.93	0.89	0.91	0.80	0.78	0.79
PLpgSQL	0.90	0.96	0.93	0.90	0.96	0.93	0.86	0.86	0.86
Perl	0.96	0.96	0.96	0.98	0.95	0.96	0.89	0.80	0.84
Pod	0.97	0.98	0.98	0.97	0.98	0.97	0.75	0.88	0.81
PowerShell	0.96	0.97	0.97	0.99	0.96	0.98	0.87	0.95	0.91
Proguard	0.99	0.97	0.98	0.99	0.98	0.99	0.99	0.80	0.88
Prot. Buffer	0.99	0.98	0.99	0.97	0.97	0.97	0.89	0.93	0.91
Pug	0.96	0.94	0.95	0.98	0.96	0.97	0.85	0.77	0.81
Python	0.80	0.78	0.79	0.82	0.74	0.78	0.60	0.54	0.56
QML	0.96	0.98	0.97	0.97	0.98	0.97	0.78	0.90	0.83
QMake	0.98	0.98	0.98	0.99	0.99	0.99	0.96	0.96	0.96
R	0.98	0.99	0.99	0.98	0.99	0.99	0.95	0.97	0.96
RDoc	0.98	0.97	0.97	0.97	0.97	0.97	0.76	0.82	0.79
RMarkdown	0.99	0.98	0.99	0.97	1.00	0.98	0.93	0.97	0.95
RPM Spec	0.99	0.99	0.99	0.98	1.00	0.99	0.97	0.94	0.96
Ragel	0.94	0.94	0.94	0.92	0.96	0.94	0.81	0.83	0.82
Rascal	1.00	1.00	1.00	1.00	1.00	1.00	0.99	1.00	0.99
ReST	0.94	0.92	0.93	0.93	0.94	0.94	0.76	0.83	0.79
RTF	0.97	0.98	0.98	0.98	0.99	0.99	0.93	0.94	0.94
Roff	0.90	0.66	0.76	0.89	0.65	0.75	0.85	0.64	0.73
Roff Manp.	0.73	0.90	0.81	0.73	0.92	0.81	0.71	0.86	0.78
Ruby	0.81	0.81	0.81	0.77	0.83	0.80	0.66	0.72	0.69
Rust	0.88	0.91	0.89	0.92	0.93	0.92	0.66	0.83	0.74
SCSS	0.75	0.78	0.77	0.77	0.82	0.79	0.60	0.70	0.64
SQL	0.88	0.69	0.77	0.69	0.67	0.68	0.75	0.53	0.62
SVG	0.73	0.80	0.77	0.82	0.85	0.83	0.87	0.66	0.75
SWIG	0.84	0.91	0.87	0.84	0.89	0.86	0.69	0.71	0.70
SaltStack	1.00	1.00	1.00	1.00	1.00	1.00	1.00	0.99	1.00
Sass	0.97	0.94	0.96	0.98	0.96	0.97	0.93	0.91	0.92
Scala	0.96	0.93	0.94	0.95	0.94	0.94	0.77	0.88	0.82
Scheme	0.94	0.99	0.97	0.97	0.99	0.98	0.90	0.97	0.94
Shell	0.88	0.91	0.89	0.89	0.93	0.91	0.62	0.64	0.63
Slash	0.99	1.00	0.99	0.99	1.00	1.00	0.90	0.99	0.94
Smarty	0.95	1.00	0.97	0.96	1.00	0.98	0.90	1.00	0.95
Starlark	0.96	0.94	0.95	0.95	0.95	0.95	0.91	0.86	0.88
Stylus	0.90	0.94	0.92	0.94	0.94	0.94	0.86	0.89	0.88
Svelte	0.88	0.98	0.93	0.89	0.98	0.93	0.78	0.92	0.84
Swift	0.89	0.90	0.89	0.90	0.84	0.87	0.57	0.48	0.52
TOML	0.98	0.97	0.98	0.98	0.98	0.98	0.93	0.95	0.94
TSQL	0.76	0.88	0.82	0.72	0.84	0.78	0.69	0.77	0.73
TSX	0.64	0.73	0.68	0.65	0.63	0.64	0.57	0.43	0.49
Tcl	0.92	0.99	0.96	0.90	0.99	0.95	0.81	0.94	0.87
TeX	0.99	0.96	0.97	0.98	0.95	0.97	0.93	0.88	0.91
Texinfo	0.99	0.99	0.99	1.00	1.00	1.00	0.94	0.92	0.93
Text	0.66	0.90	0.76	0.64	0.88	0.74	0.82	0.71	0.76
Textile	0.97	0.95	0.96	0.98	0.95	0.97	0.94	0.89	0.92
Twig	0.95	0.96	0.96	0.94	0.95	0.95	0.86	0.88	0.87
TypeScript	0.78	0.78	0.78	0.77	0.79	0.78	0.45	0.35	0.40
UnixAssembly	0.81	0.89	0.84	0.81	0.89	0.85	0.60	0.79	0.68
Vim Snippet	0.98	0.91	0.94	0.99	0.94	0.97	0.96	0.85	0.90
Vim script	0.96	0.99	0.98	0.92	0.99	0.96	0.87	0.91	0.89
VB .NET	0.99	0.94	0.96	0.96	0.96	0.96	0.95	0.90	0.93
Vue	0.91	0.84	0.87	0.94	0.86	0.90	0.76	0.74	0.75
Wavefront	1.00	0.91	0.95	1.00	0.89	0.94	0.90	0.67	0.77
WebIDL	0.99	1.00	1.00	0.98	1.00	0.99	0.94	0.99	0.97
Windows RE	0.99	1.00	1.00	0.99	1.00	1.00	0.98	0.99	0.99
XML	0.79	0.97	0.87	0.89	0.85	0.87	0.63	0.81	0.71
XML Pr. List	1.00	1.00	1.00	0.99	1.00	1.00	0.99	0.99	0.99
XSLT	0.95	0.57	0.71	0.98	0.73	0.84	0.79	0.54	0.64
YAML	0.94	0.99	0.96	0.96	0.99	0.97	0.88	0.92	0.90
Yacc	0.96	0.97	0.97	0.95	0.94	0.95	0.85	0.85	0.85
**Micro avg.**	0.92	0.92	0.92	0.92	0.92	0.92	0.83	0.83	0.83
**Macro avg.**	0.92	0.92	0.92	0.93	0.92	0.92	0.83	0.83	0.83

## Additional Information and Declarations

### Data Availability

The following information was supplied regarding data availability: A replication package for the experiments, with the code that generates the code snippet images, are available in the Supplemental Files and at Zenodo: Francesca Del Bonifro, Maurizio Gabbrielli, Antonio Lategano, & Stefano Zacchiroli. (2021). Image-based Many-language Programming Language Identification - Replication Package (Version 1.0) [Data set]. IEEE Software. Zenodo. http://doi.org/10.5281/zenodo.4986391.
